# Longitudinal Microbiome and Metabolome Shifts After Successful Intervention in Impending Stunting in Indonesian Infants

**DOI:** 10.3390/nu17223570

**Published:** 2025-11-14

**Authors:** Conny Tanjung, Ryohei Shibata, Bahrul Fikri, Titis Prawitasari, Andi Alfian Zainuddin, Aidah Juliaty, Dwi Sora Yullyana, Tonny Sundjaya, Hedi Kuswanto, Jessica Clarensia, Naoki Shimojo, Berthold Koletzko, Hiroshi Ohno, Nasrum Massi

**Affiliations:** 1 Post Graduate School, Hasanuddin University, Makassar 90245, Indonesia; conny.tanj@gmail.com; 2 Laboratory for Intestinal Ecosystem, RIKEN Center for Integrative Medical Sciences (IMS), Yokohama 230-0045, Japan; shibataryohei@gmail.com (R.S.); hiroshi.ohno@riken.jp (H.O.); 3 Department of Pediatrics, Faculty of Medicine, Hasanuddin University, Makassar 90245, Indonesia; bahrulfikriyahya@gmail.com (B.F.); aidah_juliaty@yahoo.com (A.J.); 4 Department of Child Health, Dr. Cipto Mangunkusumo National Central Hospital, Faculty of Medicine, Universitas Indonesia, Jakarta 10430, Indonesia; tprawitasari@yahoo.com; 5 Public Health and Community Medicine Department, Faculty of Medicine, Hasanuddin University, Makassar 90245, Indonesia; a.alfian@med.unhas.ac.id (A.A.Z.); hedikuswanto@unhas.ac.id (H.K.); 6 Institute of Research and Community, Microbiome Research Division, Hasanuddin University, Makassar 90245, Indonesia; yullyana.sora@gmail.com; 7 Department of Epidemiology, Faculty of Public Health, University of Indonesia, Jakarta 16424, Indonesia; s_ton77@yahoo.com; 8 Graduate School of Medicine, Chiba University, Chiba 263-8522, Japan; jessica_clarensia@yahoo.com; 9 Center for Preventive Medical Sciences, Chiba University, Chiba 263-8522, Japan; shimojo@faculty.chiba-u.jp; 10 Department of Pediatrics, LMU-Ludwig Maximilians Universität Munich, Dr. von Hauner Children’s Hospital, 80337 Munich, Germany; berthold.koletzko@med.uni-muenchen.de; 11 Department of Microbiology, Faculty of Medicine, Hasanuddin University, Makassar 90245, Indonesia

**Keywords:** microbiome, metabolome, weight faltering, Indonesian infants, stool sample analysis

## Abstract

**Background/Objectives**: Stunting and weight faltering (WF) remain pressing public health challenges in low- and middle-income countries, with long-term consequences for child growth, development, and survival. While the role of gut health in early growth is increasingly recognized, evidence on how the gut microbiome and metabolome respond to nutritional interventions in WF infants is scarce. This study explored gut microbiome and metabolome changes in Indonesian infants aged 6–12 months who overcame WF following a one-month intervention. **Methods**: Infants were assigned to either a Nutritional Advice (NA) group or a Nutritional Advice plus Oral Nutritional Supplements (NAONS) group. Stool samples were collected before and after the intervention for microbiome (16S rRNA sequencing) and metabolome (LC-MS) analysis. **Results**: Significant shifts in gut microbial composition (beta diversity) and species richness (Chao1 index) were observed in both groups, suggesting enhanced microbial diversity and gut resilience. Within-group analysis revealed increases in beneficial genera such as *Faecalibacterium* and *Peptostreptococcus*, and a reduction in pro-inflammatory Fusobacterium in the NA group. The NAONS group showed a notable decrease in *Proteus*, a potentially pathogenic genus. Between-group comparisons indicated higher abundances of *Lactococcus* and *Leuconostoc* in the NAONS group, likely reflecting the influence of milk protein-rich supplements on microbial colonization, favoring lactic acid bacteria over SCFA-producing taxa, leading to better gut health. Metabolome analysis revealed significant changes in the NA group, increases in metabolites like Threonine, Tryptophan, and Xylose pointed to improved energy metabolism and gut health, while a decrease in Oxalic Acid suggested better metabolic efficiency. In contrast, the NAONS group, while benefiting from rapid weight gain, displayed a distinct metabolic profile influenced by high milk protein intake. No significant correlations were found between microbiome and metabolome changes, highlighting the complexity of gut-host interactions, suggesting that the interventions led to independent shifts in the aforementioned profiles. **Conclusions**: Overall, the findings suggest that nutritional interventions may enhance gut health and support recovery from weight faltering, providing insights into strategies that may contribute to restoring healthy growth trajectories and preventing stunting by modulating gut health.

## 1. Introduction

Stunting and weight faltering (WF) in infants remain pressing public health challenges, particularly in low- and middle-income countries. Weight faltering, characterized by inadequate weight gain during infancy, often reflects insufficient nutrient intake and can precede impaired linear growth. These conditions are associated with long-term consequences, including suboptimal cognitive development, reduced intelligence quotient (IQ), increased susceptibility to infections, and elevated mortality risk. Moreover, stunting is largely irreversible once established, underscoring the importance of early identification and intervention [1,2,3,4].

In Indonesia, the persistently high prevalence of stunting, potentially driven by unique dietary patterns, environmental exposures, and socioeconomic disparities, highlights the urgent need for population-specific research. This study addresses that need by focusing on Indonesian infants recovering from weight faltering, offering novel insights into microbiome and metabolome responses within a Southeast Asian context. Emerging evidence from microbiome and metabolome studies suggests that gut microbial composition and metabolic profiles play a pivotal role in infant growth and development. The gut microbiome (a complex community of microorganisms residing in the gastrointestinal tract) contributes to nutrient absorption, immune regulation, and overall health. Alterations in microbial diversity have been linked to malnutrition and growth faltering. Similarly, the metabolome, encompassing all small-molecule metabolites within a biological system, provides a snapshot of metabolic activity and can reveal disruptions in pathways that contribute to growth and development [5,6,7,8].

Understanding the interplay between the gut microbiome and metabolome offers a promising avenue for identifying potential biomarkers and therapeutic targets to support healthy growth trajectories. However, research exploring these interactions in infants recovering from weight faltering remains limited.

This study aims to explore the shifts in gut microbiome and metabolomic profiles among Indonesian infants who have successfully recovered from weight faltering, a population that has been underrepresented in global microbiome research. By focusing on infants from Makassar, South Sulawesi, this study provides context-specific data that may inform locally tailored nutritional interventions. Through stool sample analysis, we aim to identify microbial and metabolic changes associated with recovery from weight faltering. These findings may inform future research aimed at developing targeted nutritional and therapeutic interventions. Building on previous studies that have linked specific bacterial taxa and metabolic pathways to improved growth outcomes [5,6], this research explores the potential of microbiome-modulating strategies—such as probiotics and prebiotics—as tools to enhance infant health and development [9].

## 2. Materials and Methods

### 2.1. Subjects

Participating infants and their families were recruited by the staff of 30 primary health facilities in Makassar, South Sulawesi, Indonesia. Eligible for inclusion were infants aged 6–12 months at the time of study entry who met the criteria shown below and whose parents provided written informed consent. Participants were included between March 2022 and March 2023.

Inclusion Criteria:

1. Infants aged 6–12 months with WF, defined as weight increments < P15th of the WHO weight increment table [10];
2. Growth chart available for monitoring (weight, length, and head circumference measured at least once at birth);
3. Available height and weight data from both father and mother;
4. A parent (either mother or father) agreed to participate in this study and signed their informed consent.

Exclusion Criteria:

1. Subjects with a length-for-age *z*-score (HAZ) below 2 SD;
2. Severe acute malnutrition;
3. Presence of cow’s milk allergy;
4. Presence of lactose intolerance;
5. Presence of galactosemia;
6. Major congenital anomaly, severe stunting at birth (newborns whose length-for-gestational age was below 10th percentile), thyroid disorder, major gastrointestinal disease, or other severe diseases, e.g., pneumonia or dehydration;
7. Conditions that require special diets, e.g., major renal or hepatic dysfunctions;
8. Conditions that influence nutritional status, e.g., moderate to severe dehydration, edema, organomegaly;
9. Infants with relative WF but a body weight above the median weight for length (considering that they may become overweight);
10. History of a low birth weight (less than 2500 g).
11. History of premature birth (born after a period of pregnancy of less than 37 weeks).

### 2.2. Study Design

This study is part of a larger study that was already published in 2024 [11]. It was originally designed as a randomized controlled trial (RCT) targeting weight-faltering infants, specifically those with weight increments below the 15th percentile. However, during the screening process, it was found that most of the identified infants had weight increments below the 5th percentile (P5) (severe weight faltering). According to Indonesian Health Ministry Regulation No. 29 (2019), infants with severe weight faltering must receive oral nutritional supplements (ONSs) to support their growth. Consequently, conducting an RCT under these circumstances would not have been ethical.

The aim of this study is to explore the shifts in the gut microbiome and stool metabolome among Indonesian infants who have overcome weight faltering after one month of intervention. The infants included in this study were divided into two groups based on their weight gain percentiles: (1) the Nutritional Advice, from now on will be called NA group (NA), consisting of infants whose weight gain fell between the 5th and below the 15th percentile and who received nutritional advice; (2) the Nutritional Advice plus Oral Nutritional Supplements, from now on will be called NAONS group (NAONS), consisting of infants whose weight gain was below the 5th percentile and who received both nutritional advice and oral nutritional supplements. All participants in this study were treated following the principles outlined in the Declaration of Helsinki. This study received ethical approval from the Permanent Medical Research Ethics Committee in Medicine and Health at the Faculty of Medicine, Hasanuddin University, under approval numbers 98/UN 4.6.4.5.31/PP36/2022 and 56/UN 4.6.4.5.31/PP36/2023. Additionally, this study was registered on clinicaltrials.gov with the identifier NCT05393934.

Stool samples were collected from all infants at baseline (before the intervention) and after one month of intervention. Samples were immediately stored at −80 °C until further analysis. DNA was extracted from stool samples using a standardized protocol to ensure consistency and reliability in the analysis. The V3–V4 region of the 16S rRNA gene was then amplified and sequenced using Illumina MiSeq technology, providing detailed insights into the microbial composition of the samples. Sequencing data were processed using R version 4.4.0 (R Foundation for Statistical Computing, Vienna, Austria) within RStudio version 2025.05.0+496. Bioinformatics processing included the calculation of alpha diversity indices (Chao1, Simpson, Shannon indices) to assess species richness and evenness, performed using the “vegan” R package. Beta diversity was evaluated using the Bray-Curtis dissimilarity index and visualized through principal coordinate analysis (PCoA) plots generated using the “phyloseq”, “ape”, and “ggplot2” packages. We used the SILVA 138 database and the QIIME2 pipeline for taxonomy classification of the 16S rRNA sequences. Taxonomic classification was performed to identify bacterial genera, providing a detailed understanding of the microbial shifts associated with the intervention.

Stool metabolites were extracted from stool samples using a methanol-based extraction method, ensuring efficient and consistent isolation of small-molecule metabolites. Following extraction, stool metabolomic profiling was conducted using liquid chromatography-mass spectrometry (LC-MS), a powerful analytical technique that provides detailed insights into the metabolic composition of the samples.

The resulting metabolomic data were then analyzed using R version 4.3.3 (R Foundation, Vienna, Austria). This analysis included the calculation of beta diversity metrics with Euclidean distance to assess differences in metabolic profiles between samples for visualizing principal coordinate analysis (PCoA) plots by using the “phyloseq”, “ape”, and “ggplot2” R packages, as well as pairwise comparisons of metabolite concentrations to identify significant changes associated with the intervention.

To ensure that the analysis focused on clinically relevant and biologically meaningful patterns, we selected the top 100 microbial taxa and the top 100 metabolites based on their median abundance across all samples. Additionally, to reduce the influence of potential contaminants and technical noise, we included only those features that were present in more than 15% of stool samples. Microbiome and metabolomic datasets are known to be high-dimensional and sparse, often containing many low-abundance or rare features that appear in only a few samples. These rare features can introduce noise, lead to spurious correlations, and obscure important biological signals. By narrowing our focus to the most abundant and consistently detected taxa and metabolites, this allows for the capture of dominant gut ecosystem members—those contributing most to compositional variation and more likely to reflect functionally relevant microbial–metabolite interactions.

The statistical significance of beta diversity metrics was assessed using PERMANOVA test by using the “phyloseq” R package. This test helped determine whether the observed differences in microbial and metabolic diversity were statistically significant. For pairwise comparisons, Wilcoxon signed-rank tests (for paired data) and Wilcoxon rank-sum tests (for unpaired data) were employed to identify significant changes in bacterial genera and metabolite concentrations, providing insights into specific shifts within the microbiome and metabolome. The results were visualized using boxplots based on median values to represent the central tendency of the data. Additionally, Spearman’s rank correlation coefficients were calculated to assess the relationship between microbiome and metabolome changes, allowing for the evaluation of potential correlations between these two aspects of gut health.

## 3. Results

A total of 1031 infants from 30 primary health facilities in Makassar, South Sulawesi, were enrolled for screening, and anthropometric measures were performed in 913 infants aged 6–12 months, of which 170 showed WF below the 15th WHO percentile of weight increment (WI) without stunting.

Of the 170 infants, only 126 were successful in overcoming weight faltering after one month of intervention; among those, 51 infants were from the NA group, and 75 infants were from the NAONS group. Their characteristics at baseline are described in Table 1.

### 3.1. Microbiome Analysis

**Figure 1:**
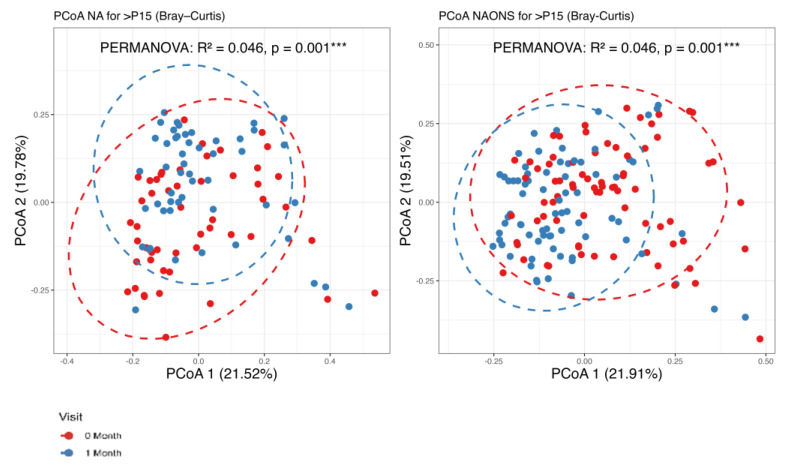
Beta diversity analysis of the microbiome of the 2 groups before and after 1–month of intervention. ***: statistically significant; PCoA: Principal Coordinate Analysis.

It was found that there are statistically significant differences observed in beta diversity of both groups (NA and NAONS groups) between the condition at baseline and 1 month after intervention, as shown in Figure 1.

**Figure 2:**
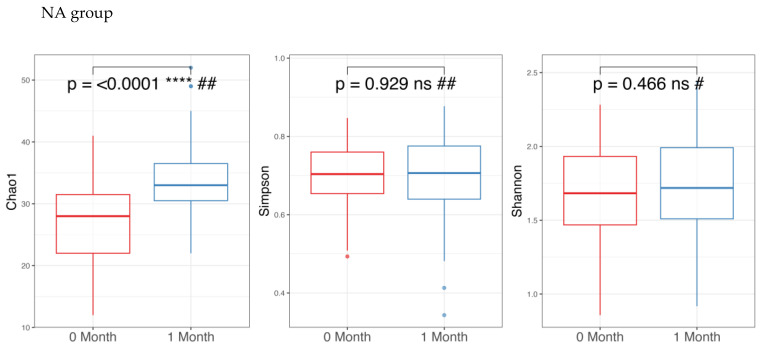

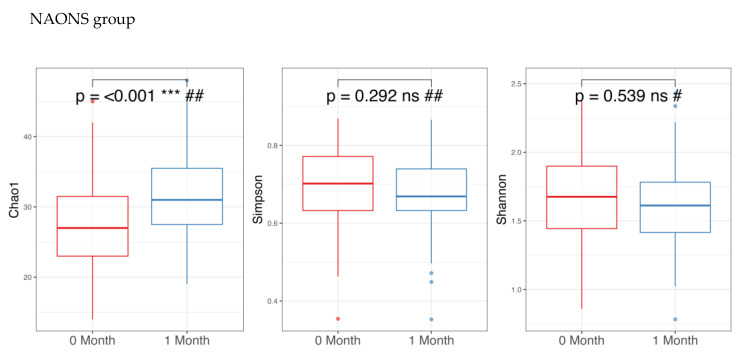
Alpha diversity analysis of the microbiome of the 2 groups before and after 1-month intervention. *** and ****: statistically significant; ns: statistically not significant; #: paired *t* test; and ##: Wilcoxon Signed-Rank test.

Regarding alpha diversity in Figure 2, only the Chao1 parameter (richness) showed a statistically significant increase (*p*-value < 0.05) in both the NA and NAONS groups, suggesting an increase in the number of different species within the gut microbiota. However, other alpha diversity indices, such as Simpson and Shannon indices, did not show significant changes.

**Figure 3:**
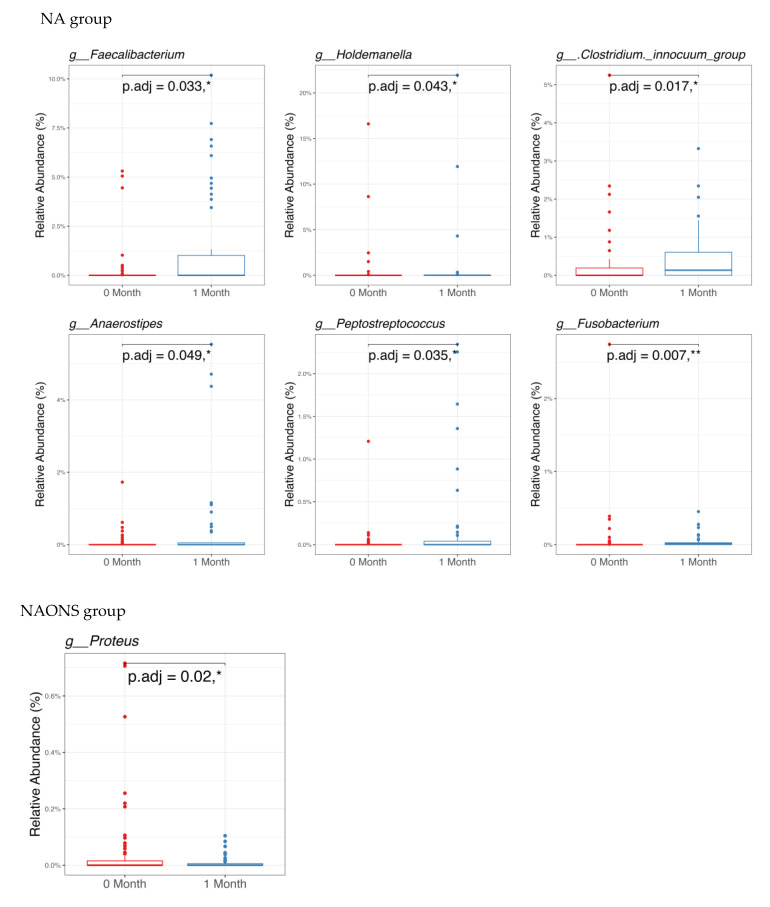
Pairwise comparison taxonomy of the 2 groups in the microbiome analysis before and after 1-month intervention. * and **: statistically significant; *p*.adj: adjusted *p*-values using Wilcoxon Signed-rank test and FDR correction.

In the pairwise comparison of taxonomy at the genus level as shown in Figure 3, the NA group showed significant changes in six genera out of the 100 bacteria with the highest relative abundance. Five genera (*Faecalibacterium*, *Holdemanella*, *Clostridium innocuum* group, *Anaerostipes*, *Peptostreptococcus*) increased, while one genus (*Fusobacterium*) decreased. In the NAONS group, out of 100 genera, two genera showed statistically significant changes, with only *Proteus* detected in at least 15% of the samples.

**Figure 4:**
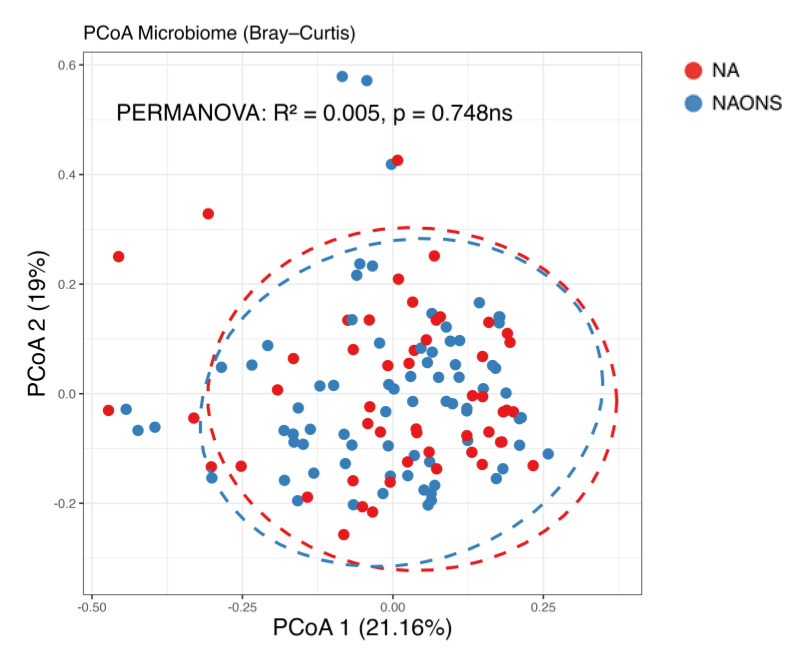
Beta diversity analysis of the microbiome of the 2 groups after 1–month intervention. ns: not statistically significant; PCoA: Principal Coordinate Analysis.

**Figure 5:**
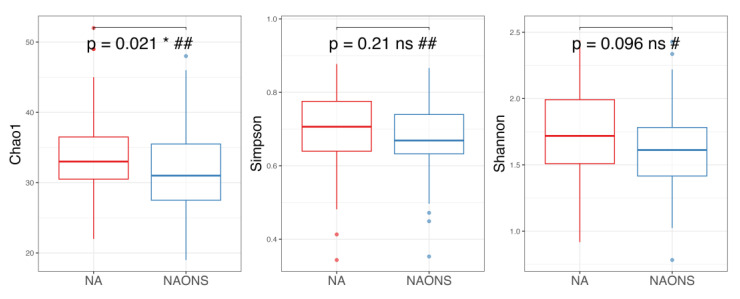
Alpha diversity analysis of the microbiome of the 2 groups after 1-month intervention. *: statistically significant; ns: statistically not significant; #: Independent *t* test; ##: Wilcoxon Signed-Rank test.

**Figure 6:**
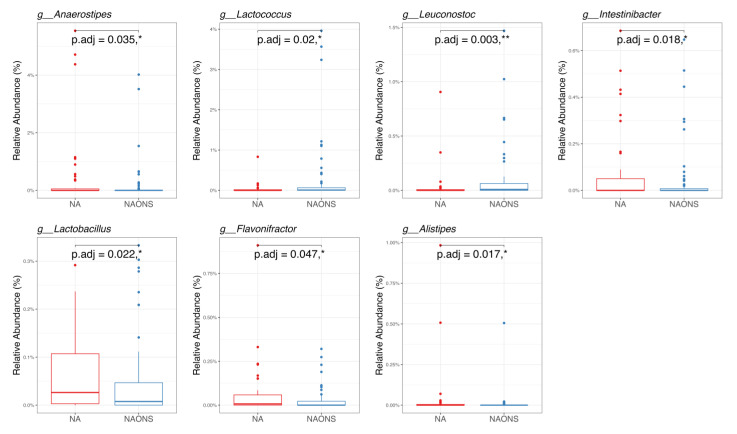
Pairwise taxonomy comparison of the 2 groups in the microbiome analysis after 1-month intervention. * and **: statistically significant; *p*.adj: adjusted *p* values using Wilcoxon Signed-rank test and FDR correction.

Given the differences in baseline weight faltering conditions between NAONS and NA groups, a comparative analysis at the end of the 1-month intervention was also performed, which is shown in Figure 4, Figure 5 and Figure 6.

In this comparison, no statistically significant differences were observed in beta diversity between the NA and NAONS groups, indicating that the overall microbial community structure was similar between the two groups after the intervention (Figure 4).

On the other hand, alpha diversity indices (Chao1, Simpson and Shannon indices) in the NA group are higher compared to the NAONS group, even though only in Chao1, it is statistically significantly higher (*p*-value < 0.05) in the NA group compared to the NAONS group. This suggests a greater microbial richness and diversity in the NA group at the end of the 1–month intervention (Figure 5).

In the genus-level taxonomic comparison, the NA group showed statistically significant differences with the NAONS group in 7 genera out of the top 100. Five genera—*Lachnoclostridium*, *Anaerostipes*, *Intestinibacter*, *Lactobacillus*, and *Flavonifractor*—were significantly higher, whereas, in two genera, namely, *Lactococcus* and *Leuconostoc*, both of which are known for their potential probiotic and gut-modulating properties, were lower in comparison with the NA group (Figure 6).

### 3.2. Stool Metabolome Analysis

**Figure 7:**
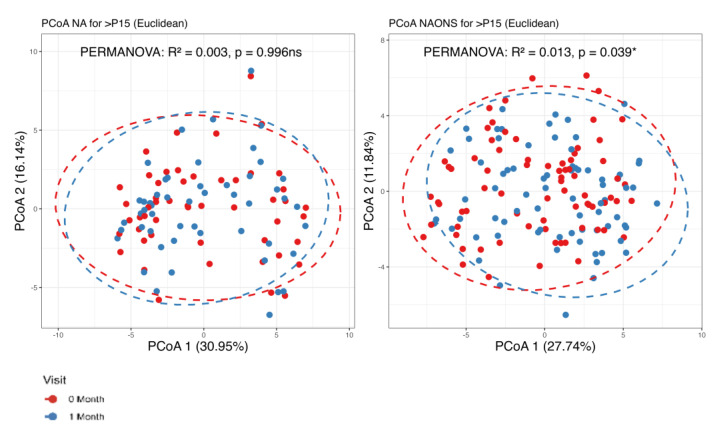
Beta diversity analysis of the metabolome of the 2 groups. *: statistically significant; ns: not statistically significant; PCoA: Principal Coordinate Analysis.

**Figure 8:**
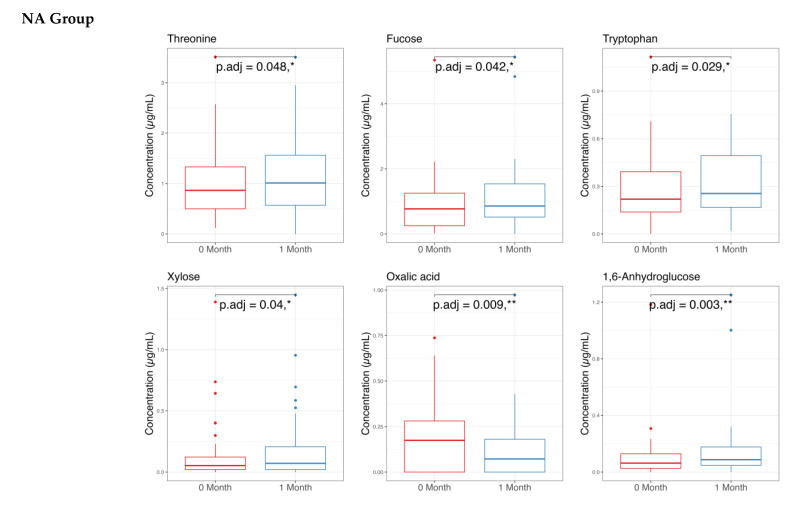

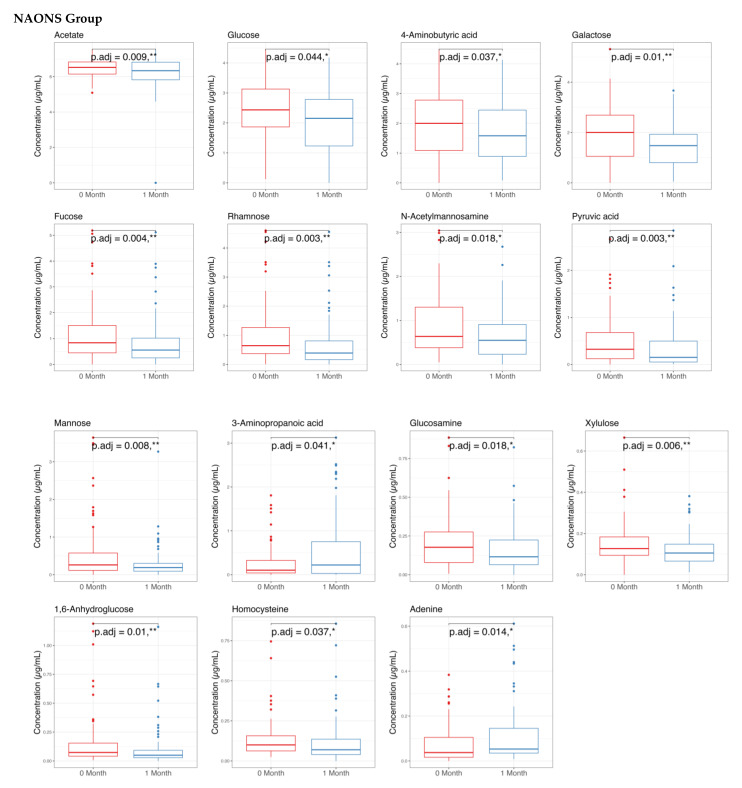
Pairwise metabolome comparison of the 2 groups in the metabolome analysis. * and **: statistically significant; *p*.adj: adjusted *p*-values using Wilcoxon Signed-rank test and FDR correction.

The metabolome analysis echoed some of the microbiome findings. In the NAONS group, significant changes in beta diversity were observed in Figure 7 (*p*-value < 0.05), indicating substantial shifts in the metabolic profile before and after the intervention. However, no significant changes were observed in the NA group. In the pairwise comparison of metabolites (Figure 8), the NA group showed significant increases in five metabolites (Threonine, Fucose, Tryptophan, Xylose, 1,6-Anhydroglucose) and a significant decrease in Oxalic Acid. In the NAONS group, two metabolites (3-Aminopropanoic acid, Adenine) showed significant increases, while thirteen metabolites (Acetate, Glucose, 4-Aminobutyric Acid, Galactose, Fucose, Rhamnose, N-Acetylmannosamine, Pyruvic acid, Mannose, Glucosamine, Xylulose, 1,6-Anhydroglucose, Homocysteine) showed significant decreases.

Additionally, no statistically significant correlations were found between the microbiota and metabolome in either the NA or NAONS groups.

**Figure 9:**
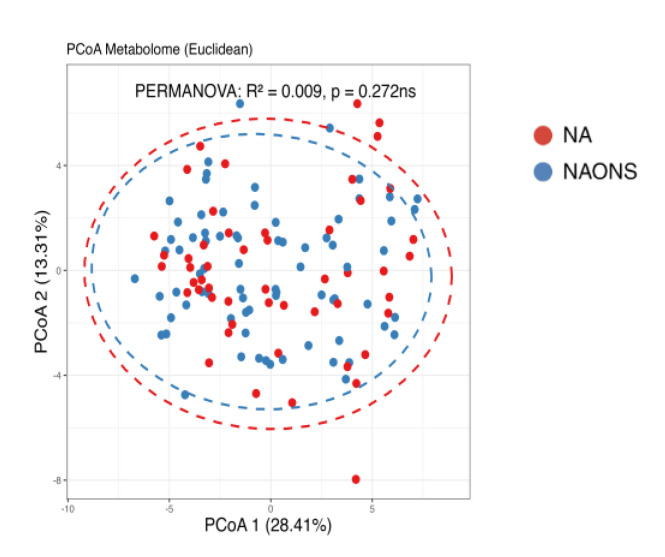
Beta diversity analysis of the metabolome of the 2 groups after 1–month intervention. ns: not statistically significant; PCoA: Principal Coordinate Analysis.

**Figure 10:**
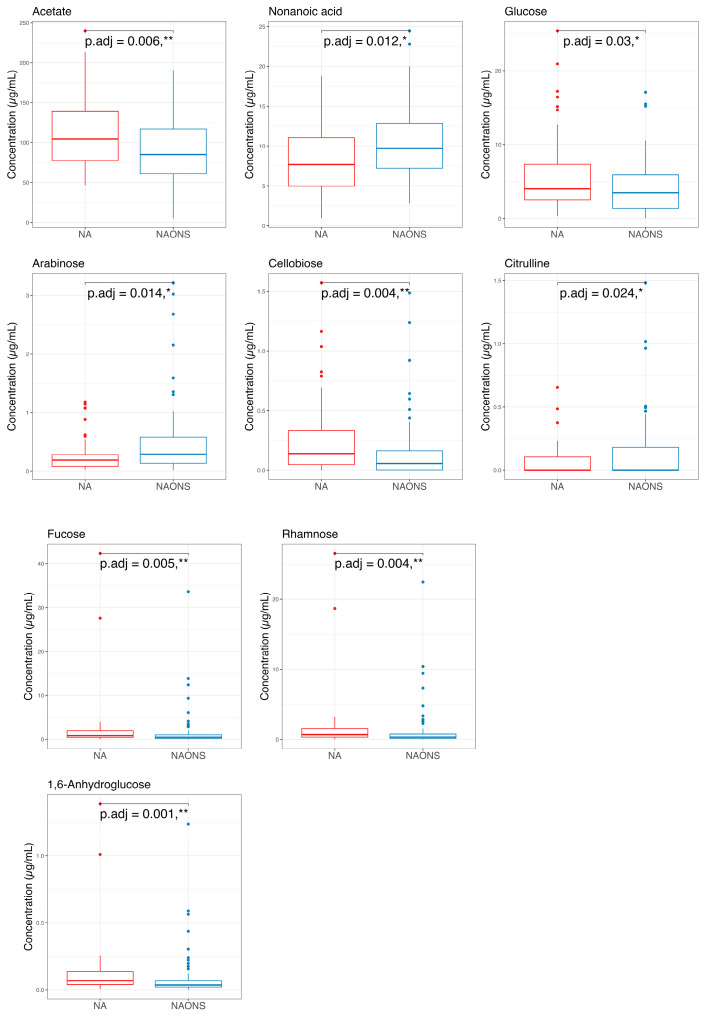
Pairwise comparison of the 2 groups in the metabolome analysis after 1–month intervention. * and **: statistically significant; *p*.adj: adjusted *p*-values using Wilcoxon Signed-rank test and FDR correction.

Finally, a comparison of the two groups at the end of a one-month intervention is also conducted because the weight faltering condition among NAONS and NA groups differs during baseline. While their beta diversity remained similar (Figure 9), suggesting comparable overall metabolic profiles, there were notable differences in specific metabolites. The NA group showed a higher level in comparison with the NAONS group in five metabolites (Acetate, Fucose, Rhamnose, Cellobiose, and 1,6-Anhydroglucose) and a lower level in four (Nonanoic acid, Valine, Isoleucine, and Arabinose) (Figure 10).

## 4. Discussion

### 4.1. On Microbiome Changes

The significant changes in beta diversity in relation to microbiome changes in both groups (NA and NAONS groups) after one month of intervention highlight the dynamic nature of gut health and its impact on growth and indicate a substantial shift in the overall microbial community structure, suggesting that the intervention led to a more diverse and potentially more stable gut microbiota. This diversity is crucial for maintaining gut health and supporting nutrient absorption [12]. And, to ensure clinical significance and avoid misleading insights due to contamination, as outlined in the methods, only the taxa that are present in more than 15% of the stool samples will be considered.

On alpha diversity, the increase in richness (Chao1) of the microbiome changes reflects a higher number of different species within the gut microbiota, which is often associated with better gut health and resilience [13].

Looking into more details in relation to the statistical changes in Specific Genera, several potential mechanisms can be the underlying process behind overcoming the weight faltering. And as mentioned above,

In the NA group,

- *Faecalibacterium*: Its elevated levels may contribute to improved gut health and integrity through its anti-inflammatory properties and the production of butyrate [14].
- *Holdemanella*: Although less extensively studied, elevated levels of this component have been associated with improved gut health [15].
- *Clostridium innocuum* group: Higher levels have been associated with improved carbohydrate metabolism and the production of beneficial metabolites [16].
- *Anaerostipes*: Increased butyrate production, resulting from higher levels, may support gut health by serving as an energy source for colonocytes [17].
- *Peptostreptococcus*: Elevated levels have been associated with enhanced protein metabolism and increased production of short-chain fatty acids [18].
- *Fusobacterium*: Often associated with inflammation and disease; its decrease suggests a healthier gut environment [19].

And in the NAONS group,

- *Proteus*: Known for its association with urinary tract infections and other inflammatory conditions, it can disrupt gut health and hinder nutrient absorption. The significant decrease in Proteus suggests a healthier gut environment, which is crucial for overcoming weight faltering [20].

At the end of this study, the NA group exhibited significantly higher abundances of SCFA-producing genera such as *Lachnoclostridium*, *Anaerostipes*, *Intestinibacter*, *Lactobacillus*, and *Flavonifractor*, particularly butyrate and acetate, which play crucial roles in maintaining gut barrier integrity and modulating immune responses [21]. Conversely, the NAONS group showed elevated levels of *Lactococcus* and *Leuconostoc*, genera often linked to dairy fermentation and probiotic supplementation. The presence of these genera may reflect the influence of milk protein-rich ONS on microbial colonization patterns, potentially favoring lactic acid bacteria over SCFA-producing taxa [22,23]. These distinct microbial profiles underscore how different nutritional interventions shape gut ecology in unique ways, with potential implications for nutrient absorption and gut function, especially in the context of rapid catch-up growth.

The role of *Lactococcus* in weight gain is an area of ongoing research, with findings indicating that its effects can vary significantly among individuals. Some studies suggest that specific strains of *Lactococcus* can enhance nutrient bioavailability, potentially contributing to weight gain in certain contexts [22]. Conversely, other strains have been linked to weight loss or maintenance by promoting a balanced gut microbiome and influencing metabolic pathways. Mechanisms through which *Lactococcus* may affect weight include modulation of gut hormones, alterations in fat storage, and changes in energy expenditure [23,24].

### 4.2. On Stool Metabolome Changes

A further deep dive on stool metabolome changes reveals that significant changes in beta diversity of the metabolome were found only in the NAONS group, indicating substantial shifts in the overall metabolic profile, reflecting the impact of the intervention on metabolic processes [25].

In NA group: The increased metabolites of Threonine, Tryptophan, Xylose, 1,6-Anhydroglucose may be due to the “cause” of becoming not weight faltering after 1-month intervention as these metabolites are involved in various metabolic pathways supporting energy production, immune function, and gut health [26], whereas the decreased Metabolite Oxalic Acid may indicate improved metabolic efficiency and reduced risk of metabolic disturbances [13,14,27].

On the other hand, in the NAONS group, the increased metabolites of 3-Aminopropanoic acid and Adenine may indicate that the potential mechanism of not becoming weight faltering is related to the 3-Aminipropanic substance, which is involved in muscle endurance and reducing fatigue [28], and adenine, which is a component of nucleotides, essential for energy transfer and cellular functions [25]. Additionally, the decrease in Acetate, Glucose, 4-Aminobutyric Acid (GABA), Galactose, Fucose, Rhamnose, N-Acetylmannosamine, Pyruvic Acid, Mannose, Glucosamine, Xylose, 1,6-Anhydroglucose, Homocysteine may add to the explanation on the positive change among weight faltering in NAONS, as these metabolites are involved in various metabolic pathways, and their decrease may indicate improved metabolic efficiency and reduced risk of metabolic disturbances [13,14,25].

Metabolomic profiling at the end of 1-month intervention revealed distinct metabolic signatures between the NA and NAONS groups. The NA group had higher levels of Acetate, Glucose, Fucose, Rhamnose, Cellobiose, and 1,6-Anhydroglucose, metabolites associated with carbohydrate fermentation and mucosal health, indicating a metabolome profile supportive of sustained gut function and growth. Notably, acetate, a key short-chain fatty acid (SCFA), supports energy metabolism in gut epithelial cells and has been associated with improved growth outcomes in undernourished children [21]. It also contributes to shaping the gut microbial environment by promoting beneficial bacteria and lowering intestinal pH, which inhibits the growth of pathogenic species. Mechanistically, acetate plays a role in maintaining immune homeostasis, supporting immune system maturation, and modulating inflammatory responses [29,30].

In contrast, the NAONS group exhibited elevated levels of nonanoic acid, valine, isoleucine, and arabinose, suggesting altered lipid and amino acid metabolism driven by high milk protein intake. These contrasting profiles underscore the differential metabolic pathways activated by each intervention. While branched-chain amino acids (BCAAs) valine and isoleucine are essential nutrients, chronically elevated levels have been associated with metabolic dysregulation [24].

The absence of statistically significant correlations between the microbiota and stool metabolome in either the Nutritional Advice (NA) or Nutritional Advice plus Oral Nutritional Supplements (NAONS) groups suggests that, while both interventions led to significant shifts in the gut microbiome and metabolome profiles, these changes did not exhibit a direct, measurable relationship. Several factors could contribute to the lack of significant correlations between microbiota and metabolome changes.

Firstly, the complexity and redundancy within microbial communities may obscure direct associations between specific taxa and metabolic functions. The gut microbiome is a highly dynamic and diverse ecosystem, where multiple microbial species can perform similar metabolic roles, leading to functional redundancy [14]. This redundancy can make it challenging to pinpoint specific microbial taxa that directly influence stool metabolite concentrations.

Secondly, the bidirectional nature of the microbiome-metabolome relationship may play a role. Stool metabolites can influence microbial growth and activity, while microbes can produce or modify metabolites [14]. This intricate interplay can result in non-linear and context-dependent associations that are difficult to capture with standard correlation analyses.

Additionally, the interventions themselves may have introduced variability that affected the microbiome and metabolome differently. For instance, oral nutritional supplements (ONSs) in the NAONS group could have provided specific nutrients that directly impacted metabolic pathways without necessarily altering the microbial composition in a predictable manner [31]. Similarly, nutritional advice (NA) may have led to dietary changes that influenced stool metabolite profiles independently of microbiota shifts.

The observed findings suggest that, while both interventions support recovery from weight faltering, the NA approach may promote a more diverse and functionally beneficial gut microbiome, which may support gut health. Meanwhile, the NAONS group’s profile, shaped by high milk protein intake, may favor rapid weight gain but with a distinct microbial and stool metabolic signature that warrants further study to validate it. These findings are aligned with recent multi-omics studies showing that early-life nutrition significantly shapes the gut microbiome and stool metabolome, with long-term implications for growth and development [30]. Importantly, the gut microbiome’s role in mediating the effects of nutritional interventions underscores the need for context-specific strategies, particularly in low- and middle-income countries where undernutrition is prevalent [21].

Findings from this study align with previous research that has demonstrated the critical role of gut microbiota in child growth and development. For instance, a study by Gough et al. found that linear growth faltering in infants was associated with specific changes in the gut microbiota, including an increase in beneficial bacteria like *Faecalibacterium* [15]. Additionally, the observed stool metabolome changes are consistent with studies that have shown the importance of amino acids and other metabolites in supporting growth and metabolic health [18].

Weight faltering and stunting are interconnected conditions that share common underlying mechanisms, such as inadequate nutrient intake and poor gut health. The significant changes in both microbiome and stool metabolome profiles observed in our study suggest that interventions targeting gut health can effectively address weight faltering and potentially prevent stunting. The increase in beneficial bacteria and essential stool metabolites indicates improved nutrient absorption and metabolic efficiency, which are crucial for healthy growth [32].

Our findings support the use of nutritional interventions that focus on enhancing gut health to address weight faltering, particularly in Southeast Asian settings where dietary practices and environmental exposures differ markedly from those in Western populations. This study contributes novel evidence to guide precision nutrition strategies tailored to Indonesian infants. Strategies such as providing oral nutritional supplements (ONSs) and tailored nutritional advice (NA) can help restore a balanced gut microbiota and improve metabolic processes. These interventions should be integrated into community health programs to ensure early identification and management of weight faltering in infants [7].

Despite observing significant shifts in both microbiome and metabolome profiles following the intervention, our study did not identify statistically significant correlations between specific microbial taxa and metabolite changes. This limits our ability to draw direct causal inferences regarding microbiota–metabolome interactions. However, this finding underscores the inherent complexity of gut–host dynamics, especially in the context of early-life nutritional interventions. The gut ecosystem exhibits functional redundancy, where multiple microbial taxa can produce similar metabolic outputs, potentially obscuring direct associations in correlation analyses.

These insights highlight the need for future studies employing integrative multi-omics approaches (such as metagenomics, metatranscriptomics, and targeted functional assays) to unravel the mechanistic pathways linking microbial shifts to metabolic outcomes. Such approaches will be essential to deepen our understanding of how specific microbial functions contribute to growth recovery and to identify actionable targets for precision nutrition strategies in undernourished populations.

This study has several limitations. First, the sample size was relatively small, which may limit the generalizability of the findings. Second, this study was conducted in a specific geographic region (Indonesia), which may reduce applicability to populations with different dietary habits and environmental exposure; this also represents a key strength. By focusing on an underrepresented Southeast Asian population, this study provides valuable, context-specific insights into gut microbiome and metabolome responses, which are critical for developing culturally and regionally appropriate nutritional interventions. Third, this study did not account for potential confounding factors such as socioeconomic status and maternal health, which could influence the outcomes [33].

Future research should focus on larger, multi-center studies, with a more robust study design (such as RCT double-blinded) to validate the findings and explore the long-term effects of nutritional interventions on growth and development. Additionally, studies should investigate the specific mechanisms through which gut microbiota and stool metabolites influence growth, including the role of the gut–brain axis and immune system. Importantly, future work should incorporate functional validation approaches, such as metatranscriptomics, metabolite flux analysis, and targeted microbial assays, to confirm causal relationships and biological relevance. Further research is also needed to develop personalized nutritional interventions that consider individual variations in gut microbiota and metabolic profiles [9,34].

## 5. Conclusions

This study examined changes in the gut microbiome and stool metabolome of Indonesian infants who overcame weight faltering following a one-month nutritional intervention. Significant shifts were observed in microbial diversity and metabolic profiles, including increased alpha diversity and changes in key bacterial genera such as *Faecalibacterium* and *Fusobacterium*. Metabolomic analysis revealed elevated levels of essential amino acids and other metabolites linked to growth and gut health.

These findings highlight a potential association between gut microbiota and metabolism in supporting recovery from weight faltering and provide valuable insights for developing effective public health interventions and clinical practices to support healthy growth and development in infants. They also underscore the importance of early-life nutrition strategies that promote gut health, particularly in low- and middle-income settings. Integrating such interventions into community health programs may help improve growth outcomes and reduce the risk of stunting.

The implications of these findings for public health and clinical practices are substantial. Nutritional interventions that focus on enhancing gut health, such as providing oral nutritional supplements (ONSs) and tailored dietary advice, may address weight faltering and contribute to stunting prevention. These interventions should be integrated into community health programs to ensure early identification and management of weight faltering in infants. By improving gut microbiota and metabolic profiles, these strategies can lead to better nutrient absorption and overall growth, reducing the risk of long-term developmental issues.

## Figures and Tables

**Table 1:** Participant characteristics at baseline.

Variable	Category	NA		NAONS		*p*-Value
		*n*	%	*n*	%	
About Siblings	Without Sibling	14	22.22	15	15.00	0.240 ns#
	Siblings	49	77.78	85	85.00	
Parity	<3	37	58.73	43	43.00	0.036 *##
	≥3	26	41.27	57	57.00	
Childbirth History	Sectio Caesaria	15	23.81	24	24.00	0.978 ns#
	Spontan	48	76.19	76	76.00	
Gender	Male	31	49.21	43	43.00	0.270 ns##
	Female	32	50.79	57	57.00	
Ethnic	Bugis	10	15.87	16	16.00	0.701 ns#
	Makassar	47	74.60	78	78.00	
	Other	6	9.52	6	6.00	
Smoking Status	Non-Smoking	24	38.10	32	32.00	0.264 ns##
	Smoking	39	61.90	68	68.00	
Income	<IDR 3,000,000	39	61.90	59	59.00	0.420 ns##
	≥IDR 3,000,000	24	38.10	41	41.00	
Mother Education	No Education At All					0.747 ns#
	Elementary School					
	Junior High School					
	Senior High School					
	Diploma 1					
	Diploma 2					
	Bachelor					
	Post-Graduate					
Father Education	No Education At All					0.758 ns#
	Elementary School					
	Junior High School					
	Senior High School					
	Diploma 1					
	Diploma 2					
	Bachelor					
	Post-Graduate					

Comparison test: Chi-square test ‘#’; Fisher exact test ‘##’; significant codes: <0.05 ‘*’; not significant ‘ns’.

## Data Availability

The original contributions presented in this study are included in the article; further inquiries can be directed to the corresponding author.
